# Access to nutritious food, socioeconomic individualism and public health ethics in the USA: a common good approach

**DOI:** 10.1186/1747-5341-8-16

**Published:** 2013-10-29

**Authors:** Jacquineau Azétsop, Tisha R Joy

**Affiliations:** 1 Département de Santé Publique, Faculté des Sciences de la Santé de l’Université de N’djaména, Avenue Mobutu, B.P. 1117 N’djaména, Tchad; 2 Department of Medicine, Western Ontario University, B5-107, 268 Grosvenor Street, London, Ontario N6A 4V2, Canada

## Abstract

Good nutrition plays an important role in the optimal growth, development, health and well-being of individuals in all stages of life. Healthy eating can reduce the risk of chronic diseases, such as heart disease, stroke, diabetes and some types of cancer. However, the capitalist mindset that shapes the food environment has led to the commoditization of food. Food is not just a marketable commodity like any other commodity. Food is different from other commodities on the market in that it is explicitly and intrinsically linked to our human existence. While possessing another commodity allows for social benefits, food ensures survival. Millions of people in United States of America are either malnourished or food insecure. The purpose of this paper is to present a critique of the current food system using four meanings of the common good--as a framework, rhetorical device, ethical concept and practical tool for social justice. The first section of this paper provides a general overview of the notion of the common good. The second section outlines how each of the four meanings of the common good helps us understand public practices, social policies and market values that shape the distal causal factors of nutritious food inaccessibility. We then outline policy and empowerment initiatives for nutritious food access.

## Introduction

A significant number of people in the United States of America (USA) are confronted with food insecurity [1-3]. Food security refers to “a situation that exists when all people, at all times, have physical, social and economic access to sufficient, safe and nutritious food that meets their dietary needs and food preferences for an active and healthy life” [4]. Lack of accessibility to healthy food is an important aspect of food insecurity, as good nutrition plays an important role in the optimal growth, development, health and well-being of individuals in all stages of life. Healthy eating can also reduce the risk of chronic diseases such as heart disease, stroke, diabetes and some types of cancer. The economic cost of food insecurity is about $90 billion per year in increased medical care costs [5]. Access to food is a “problem of special concern for women and children. Female-headed households had a food insecurity prevalence of 30.2%, or almost 3 times the national average, and more than 12.4 million children experienced food insecurity in 2007” [4]. With such a number of children confronted with food insecurity, “sizable segments of the population are at risk for poor development and impaired performance in school” [5]. The impact of food insecurity on children needs to be considered as an issue that may diminish national productivity. Other consequences of food insecurity include the loss in educational attainment and worker productivity, as well as an investment burden into the emergency food system [5].

We intend to rely on the common good approach as an innovative and valuable lens to critically understand and address healthy food insecurity and its public health consequences in the USA. The notion of the common good has been central to conceptions of society from Plato, Aristotle, Cicero, Augustine to Thomas Aquinas [6-8]. Its most basic meaning refers to the way the community and its institutions treat citizens. This notion can be understood as a set of conditions that favor human flourishing [9]. In this paper, we will refer to the common good as an analytical tool, an accusatory-rhetorical device, a normative concept and a practical tool that may possibly shape the search for a better understanding of the origin of and concrete solutions to nutritious food inaccessibility. This paper relies on these four possible usages of the common good to show that inaccessibility to nutritious food is less an issue of an individual’s inability to access quality food than a problem of structural injustice.

## The common good tradition

The common good refers to “the set of conditions that facilitate social living by which persons or groups are enabled to more fully and readily achieve their goals and perfection” [9,10]. It describes a specific “good” that is shared by and beneficial for all or by most members of the community. Due to the divergence of interests between individuals, the role of the state or community is important for the promotion of citizens’ welfare. By playing its welfare-enhancing function as well as its regulatory role, the state ensures that human rights are respected and individuals are free to participate in the state’s affairs. The public provision of these key dimensions of well-being ensures public participation in the state’s affairs and guarantees a certain level of social justice.

Approaches to the common good are not homogenous and, at times, can be antagonistic. David Hollenbach and Michael Novak, two important common good thinkers, clearly depart from each other. While Hollenbach defends an approach to the common good which is rooted in a communitarian notion of society based on the social nature of the human person [8], Novak develops a libertarian approach to the same notion based on his understanding of the human person as an individual endowed with the capacity for insight and choice, from which derives the principles which are the bases for human dignity [11]. The contemporary debate on the common good navigates among liberal, communitarian and egalitarian views of a just society.

Thomas Hobbes’s view of political society was based on a negative perception of human nature. Hobbes argued that human behavior is inclined to anarchy, and therefore, he advocated for a strong central authority which could be the guarantor of the law [12]. John Locke did not really believe in the common good *per se*, but rather approached it as definable in terms of private property [13]. Subsequently, the enlightenment moved from the idea of the common good based on human nature to a contractual understanding of social life. Jeremy Bentham argued that the meaning of the common good was derived from a notion of private interests [14]. In the same line of thought, Adam Smith did not see the common good as the sum total of private interests. He understood moral persuasion to be the only way of solving the injustices that could result from the consideration of private interest as priority [15].

Modernity brought about a conception of personhood that appears to be a threat to social cohesion and the common good. The fragmentation of the modern world has been expressed in terms of the decrease in social capital [16], failure in social integration [17] and weak sense of community and civic engagement [18]. The dominant approach to the common good in contemporary USA is essentially libertarian, stemming from the Anglo-American political tradition grounded in private property and liberal citizenship [12]. In human society, there is not only a responsibility not to harm the individual or public interest [19], but also the moral obligation to remedy social evils and overcome the problems that create a threat to human survival, no matter who has caused them. Even though there are rights pertaining to individuals, their realization often requires a common ground without which it cannot be achieved. The obligations that we have as a society are preconditions to the fulfillment of human flourishing. The common ground that society promotes and institutionalizes prevents fratricidal individualism and promotes genuine solidarity which is at the service of social cohesion and communal welfare. The future of society is at stake when we claim that we are bound by ends and roles we have chosen while our daily experience shows that ends we have not chosen shape our behaviors and determine public practices [18]. The widespread notion of liberal citizenship does not fully capture the veracity of human existence. Our choices are determined not simply by our own will but also by our identities as persons caught up in religious, family, neighborhood and other civil society networks [18]. The common good offers a space of relationality, where personal and social goals can be achieved justly. Individual autonomy and communal ventures are not mutually exclusive, but rather mutually enriching and intertwined. Underneath the common good tradition resides a fundamental attempt to reconcile personal interest and communal goals. The individual is a member of a moral community that sustains his or her life and in which he or she should participate fully. While society provides individuals with the social conditions for flourishing, individuals contribute to building a society where all members can flourish and prosper. This is done through various mechanisms that promote social and contributive justice.

The approach to the common good that will be advocated in this paper is fundamentally social and institutional and does not begin with the libertarian emphasis on the individual. Instead, our approach emphasizes individual dependence and social interdependence. It stresses the welfare-enhancing role that public institutions should play in human societies in order to include those who are economically and politically marginalized. Asserting that there are goods which are common to members of society presupposes the recognition of sociality as an important dimension of human existence. Human society is not an atomistic entity but essentially a relational one. Society is a space where mutuality and interdependence shape human relationships and public institutions. Justice is not simply understood in terms of individual good and civil-political liberties but also in terms of structural equity and well-being understood at the population level. Hence, there is a moral relevance to the affirmation of the inherent sociality of the human person, since individuals are by necessity embedded in a social network that provides goods for the benefit of each member of society. Although sociality is both a choice and necessity, it is more a necessity than a choice, because human beings are finite beings. The achievement of personal goals and the satisfaction of an individual’s needs often transcend the strength of an individual person. Public institutions’ intervention or individual support is often needed to support those who lack ability to live a decent life. The human person by himself or herself has limitations that demand integration into a larger web without which the attainment of a full life would be impossible.

The common good is a good which is applied to a human community. This community can be the family, professional groups, a social institution, and a national or an international community. The common good tempers individualism and all sorts of partisan interests that do not seek to achieve the goals set by society [20]. However, the need for constructing society should not undermine the dignity of the person and interfere with human rights. The institutionalization of a genuine solidarity can be implemented in such a way that individual welfare and liberty are not undermined. As a contestation to totalitarian misuse of political power by the Nazi regime, Jacques Maritain clearly stressed that in no way would the common good hinder the good of the human person [21]. He argues that the person transcends the social order and is ordained to the society of divine persons; the common good enables progress toward absolute goods [21]. The population-based dimension of health promotion, as well as the importance accorded to the community by health promoters, gives a prominent place to the common good language in public health. This notion can be referred to as “the welfare of individuals considered as a group” [22]. He draws his views on traditions which see the good of society as more than the sum of individual goods, and where that good is expressed through practices.

### The common good as an analytical framework: framing healthy food inaccessibility as a public health issue

Framing, in public health discourse, refers to the process of evaluating a particular problem for the purpose of developing policy recommendations and an adequate intervention. Social justice is often used as a prism through which evaluation is done and intervention carried out [23]. In the present case, this framing process can help uncover the causes and consequences of healthy food inaccessibility in some population groups.

When framed within the common good perspective, inaccessibility to quality food questions the degree of social cohesiveness in the USA. There is something morally disruptive about individualism, because it does not seem to promote the goods which are common to society. The common good challenges us to highlight how the lack of social cohesiveness [22], the progressive erosion of social capital and social relationships [16], prevents the institutionalization of a genuine solidarity [24]. The causes of healthy food inaccessibility are essentially structural. The recourse to the common good helps frame this issue in such a way that we come up with “the fundamental cause” [25]. The concept of fundamental cause “involves resources like knowledge, money, power, prestige, and social connections that determine the extent to which people are able to avoid risks for morbidity and mortality” [26]. As for the common good, the concept of “fundamental cause” emphasizes the need for contextualizing the reasons for poor diet and an individual’s inability to access healthy food by examining what puts people at risk of risks and what healthy food inaccessibility means to Americans as a nation. The same concept points to the fact that socioeconomic status and the lack of a genuine institutional support are likely to be the “fundamental causes” of healthy food inaccessibility because “they embody access to important resources, affect multiple disease outcomes through multiple mechanisms, and consequently maintain an association with disease even when intervening mechanisms change” [26]. Policy-makers and health professionals should pay attention to these possibilities in order to adopt broad-based societal interventions that could improve people’s access to quality food and reduce the burden of disease [26].

The association of diets high in fat, salt, and sugar with the development of obesity, type 2 diabetes (DM2), dyslipidemia, and cardiovascular disease (CVD) is well-documented [27-29]. The 2010 Dietary Guidelines for Americans focus on dietary quality, emphasizing consumption of fruits and vegetables, low-fat dairy products, and whole grains [30]. National efforts to increase healthy food consumption have included health promotion campaigns aimed at educating individuals regarding the dietary guidelines and reading nutrition labels. Moreover, meeting these dietary recommendations often proves difficult, particularly among low-income adults and certain ethnicities such as First Nations and non-Hispanic blacks [31-35]. Although cost and availability are important constraints to healthy food accessibility, addressing these barriers may be overlooked when prescribing lifestyle intervention or solutions based only on government provision for food stamps. Altering access barriers through policy may improve individual ability to choose a good lifestyle.

Food insecurity is not simply an expression of an involuntary absence of food, rather, that poor diet can result from entitlement failure [36]. Access to adequate nutrition is influenced by structural and socioeconomic arrangements that prevent individuals from acquiring basic capabilities to function as agents of their own destiny [36,37]. Mariana Chilton and Donal Rose highlighted the social dimension of food insecurity. These authors considered food insecurity as an “outcome of social and economic processes that lead to lack of access to food. These are: lack of adequate education and living wages, lack of access to health care and health information, and exposure to unsafe living conditions such as unsafe water, poor housing, and dangerous neighborhood environments. Each of these is recognized to be integrally associated with poverty” [5].

Within the common good frame, food inaccessibility is amenable to broad policy solutions when the determinants of accessibility are rethought in systemic terms. The common good framework is not an individualizing frame. Individualizing frames limit the causes of a problem to particular individuals, often those who are afflicted with the problem [23]. Instead, the common good broadens the focus, assigning responsibility for poor diet and its public health consequences not only to individuals but also the government, corporate business and larger social forces [23]. Since the common good refers to both social and personal realities, it includes both systemic and personalizing elements, even though the systemic pole tends to be dominant.

### Common good as an accusatory concept

As an analytical tool, the common good helps interpret empirical facts of socioeconomic marginalization and their impact on individual well-being and on society. The economic and human cost of healthy food insecurity cannot be underestimated. A socially grounded analysis allows us to understand the connection between the fact of exclusion and the impact of food insecurity on individuals and communities. When the freedom to choose healthy food is constrained by factors beyond consumer control, it is morally unjustifiable to hold an individual accountable for the health damages caused by poor diet. The common good unveils the economic and health consequences of uneven access to nutritious food.

Socioeconomic status has been inversely linked to type 2 diabetes (DM2) [38] and cardiovascular disease (CVD) risk [39,40], and low-income consumers have cited unavailability and cost as constraints to healthy eating, thereby participating in behaviors that may be ultimately detrimental to their health [41]. Typically, healthy diets are more expensive than unhealthy ones [42-45]. Social barriers to food accessibility include crime and poverty which may not only deter residents from walking to local grocery stores but also may impede chain supermarkets from locating in these areas [46]. Consequently, geographically-isolated populations or low-income neighborhoods may be served only by smaller grocery stores, which often have limited access to healthy foods [47]. Even when these products are available, a recent US study demonstrated that transforming the recommended standard food basket (based on the US Department of Agriculture’s Thrifty Food Plan) to a healthier alternative resulted in an additional 17-19% in cost, equivalent to 35-40% of a low-income consumer’s food budget [47]. Although state-based food assistance programs are in place in the USA, these have been demonstrated to be either inadequate for covering the cost of purchasing healthy foods or variably accepted by stores [48,49].

The consequences of poor diet resulting from food insecurity are significantly important. Even when income and educational status are controlled for, food insecurity is “associated with poor health status in children and adults, depression and anxiety among adolescents and adults and adolescent suicidal ideation, even the mildest form of food insecurity is associated with risk of poor cognitive, social, and emotional development of children younger than 3 years in the USA” [5]. Food-insecure households are confronted with “lower nutrient intakes, poor child development, poor health, and forced trade-offs between paying for basic needs such as housing, heating, and medical care. Each trade-off situation increases vulnerability” [5]. Those who already carry the burden of social and/or geographical marginalization become further victims of economic discrimination and market tactics that do not promote their health.

The common good framework offers the possibility of highlighting the moral significance of community as a space for mutual care and reciprocity. A call for individual responsibility for poor eating and its health consequences becomes less significant for people living in neighborhoods where access to healthy food represents a real challenge. Lasting changes incorporating the common good argument entails more than a call for behavior change and mere charity. It calls for change in culture and environment to support behavior change. Though mere distribution of healthy food is not the solution either—it can be a transitional solution. Instead, a change in the mindset and public practices that determine the configuration of the food environment and patterns of social relationships is much needed. The individualistic mindset that shapes social institutions and risk perception should be questioned because, with such an ideology, issues of food security and their health consequences pertain to individual responsibility especially for those who live where healthy food is not accessible.

Disparities in food insecurity rates have not changed since 1998: “African American and Latino households continue to have 2 to 3 times the prevalence of household food insecurity compared with White households. It has been well documented that geographic disparities also exist in access to healthy foods” [5]. A common good approach entails focusing on those who are most vulnerable, understanding what causes this vulnerability or susceptibility to adverse outcomes, and changing conditions to improve their situation.

Poor diet with consequent obesity can lead to the increase in chronic diseases. Obesity generates increased health-care costs and reduced economic productivity. Looking at obesity in terms of annual health-care costs, we discover that “obesity accounts for US$75 billion, half of which is paid by the Medicare and Medicaid programs. Health-care spending on obesity accounted for more than a quarter of the rise in per-capita health-care spending from 1987 to 2001” [25].

### Common good as an ethical concept

The common good is a normative concept because the act of framing directly or indirectly implies a judgment of value. The “good” we are talking about is that which perfects a human person as well as social institutions. The common good perfects, achieves, and completes a person as a relational and rational being. Rethinking healthy food access cannot be done without judging the level of justice in society and questioning the public values that shape the official discourse on food access. Social inequalities generated by individualism and justified by appeals to the self-regulatory market argument are not justifiable in a country where some have far more than what they actually need and others go without the minimum required to be healthy. Quality food inaccessibility affects the individuals and population groups and reflects what goes on in society. Inaccessibility points to the way individuals relate with each other and social institutions. It also shows how dimensions of well-being are distributed and how government fulfills its duty as public health protector and social justice promoter. Inaccessibility raises both human rights and social justice questions. Justice requires that those lacking healthy food be provided with the basic dimensions of well-being which are instrumental for having access to it [37].

#### Access to food as a basic right

Access to healthy food is a right, not a privilege to be enjoyed by few. This right is usually inherent in the right to an adequate standard of living for health and well-being [50], where food adequacy refers to both food quality and food quantity. It is one of those rights that provides an individual with the ability to function properly in order to participate in society’s affairs [51]. For us, this right should be explicit in its provisions to ensure people with the important dimensions of well-being. The constitutional law of countries that ratified the Universal Declaration of Human Rights (UDHR) acknowledges that the state has to guarantee adequate living conditions for everybody [50]. Whenever individuals or groups are unable, for reasons beyond their control, to obtain adequate food through the means at their disposal, states have the obligation to fulfill that right directly.

Although food is necessary for human survival, individual purchasers are reduced to consumers within a market, rather than humans seeking subsistence. As a result, food is categorized as a commodity available to consumers with purchasing power rather than as a universal human right. Food is purchased and sold by individuals under free competition and is subject to the “invisible hand” of the market [15]. The capitalist mindset that shapes the food environment has favored the commoditization of food. Food is just a marketable commodity like any other commodity. However, food is different from other commodities in the market as it is explicitly and intrinsically linked to our human existence. While many other commodities confer social benefits, food ensures survival.

National governments have the duty, under international law, to respect, protect, and fulfill the right to adequate food. The right to food is grounded in the respect for equality between persons, which is affirmed as the foundation for all human rights [51]. The concept of equality is not just a conclusion derived from philosophical premises, but refers to the absolute necessity to consider a fellow human being always as an end and never as a means to any social endeavor [51]. The reification of human dignity is fundamentally unjust from the standpoint of international law; fundamental equality, stated by the preamble of the UDHR [52], is at stake when some people are marginalized and do not have access to health-promoting foods. Having the basic material goods to sustain a healthy life is an expression of respect for the dignity of each person. Equality requires institutional solidarity which is not a matter of charity, but essential to what means to be being a good and healthy society. The human rights obligation to improve healthy food accessibility requires national stakeholders and the global community at large to advocate for and to implement strategies “that are comprehensive in their scope and coordinated in their implementation. Such strategies must be linked to pro-poor initiatives which include concrete actions for the various duty bearers [53].” As an important entitlement, the right to food questions the market philosophy that promotes socioeconomic exclusion and disregards the claims of the poorest among the poor. Access to quality food is an important dimension of the right to food. Hence, “it also allows claimants to assert and claim their rights, making the critical shift from treating hunger and food insecurity as a charitable endeavor to recognizing adequate food as a right that must be protected by law [53].”

#### Access to food, an issue of social justice

Poor diet and ill health are causally connected due to constraints imposed by structural inequality rather than individual failure—that is, a social justice versus a victim-blaming explanation. Social justice is concerned with what society, as a whole, owes to its individual members [22]. Every approach to justice implies an existing common ground that binds individuals together for some common causes including health promotion, respect for private property, national security, promotion of the common interest of citizens and so on [22].

Our approach to justice focuses on the well-being of people in social communities and groups because reliance solely on distributive principles cannot be the bedrock for solving nutritious food inaccessibility in isolation from larger issues of social equity that shape the overall context of well-being in the USA [37]. This approach includes many dimensions of well-being of which each represents something of independent moral significance [37]. It provides tools to show how access differentials to healthy food across populations can be understood as resulting from the lack of important dimensions of well-being in marginalized population groups. Compared to men, women’s access to food is constrained by factors beyond their control.

It is argued that “Female-headed households have a high prevalence of household food insecurity—30.2% compared with 11.1% in the general US population” [5]. A woman who is a head of a low-income household may be vulnerable to food insecurity because she lacks many dimensions of well-being, including decent income and childcare support compared to a woman who is married and educated and has adequate skills. The low-socioeconomic status of single woman who is a head of a family may be related to the fact that she has less education, fewer skills, less access to higher paying jobs, and, thus, more stress and anxiety about affording food [5]. Similarly, the facts of vulnerability to food insecurity and the risks of poor health that she faces in her life shows that such a woman lacks basic dimensions of well-being. Most single-headed households and other vulnerable individuals or groups are faced with social exclusion and lack of social support that reduce their ability to participate in the market and other social ventures. The access to healthy food emerges as a requirement of justice, based on the uncompromised worth that every human enjoys by the fact of being a human being. It is not given to someone by another person; instead, it is a quality one enjoys by virtue of being part of the human family [8,9,20,51]. Whatever interferes with human worth cannot be tolerated.

### The common good as a practical tool

How and in which policy domains can we translate our normative concerns into concrete proposal for action? Ethical reflection is not a mere rhetorical enterprise. The common good cannot be reduced to a rhetorical, normative and critical device with no influence on practical matters. Instead, it should serve as a tool to identify practical solutions which are informed by real world realism and the structural etiology of poor eating. In this regard, it can be a means for socially grounded decisions and policies that challenge socioeconomic and geographic barriers to quality food. An ethical reflection fulfills its purpose when it moves from rhetoric to the realm of social activism. There is no ethics without action. Analyzing quality food inaccessibility through the lens of the common good cannot be limited to a mere act of denunciation. It points to the need for solidarity rooted in a lasting social change and institutionalized through social policy. Lasting initiatives rooted in solidarity should include advocacy and the empowerment of those who cannot have access to healthy foods by themselves.

#### The need for a genuine institutional solidarity

One of the major problems with the common good approach as a tool for justice lies in its opposition to the dominant moral language in the USA. The conception of moral life gravitates around the individual and not the community. Making the case for the common good is somewhat challenging given the ideological roots of policy preferences in the USA [25]. The prevailing political ideology perceives the common good as being opposed to individual autonomy which provides freedom to have access to whatever one wishes, as long as one’s choices do not harm others [10,25]. An ongoing challenge in the history of public health in the USA has been the struggle between the rights of the autonomous individual versus the needs and goals of the community. The individual autonomy pole of the struggle is praised by most Americans while that of the community tends to be undermined [22,23,25]. This cultural resistance to communitarian values reinforces the political resistance of powerful entities that could be targeted for blame and made to bear some burden in the solution [25]. Governmental regulation designed to proactively shape the food environment therefore involves a serious battle for political and popular support.

Understood from the standpoint of moral and political liberalism, the rights language predominantly conveys the core of moral life in the USA. Dan Beauchamp distinguishes the first language and the second language of moral life in the USA [22]. The first language stems from the constitutional-republican conception of liberty and civil-political rights of individuals while the second language stems from the American communitarian heritage [22]. Beauchamp emphasizes that “Public health as a second language reminds us that we are not only individuals, we are also a community and a body politic, and that we have shared commitments to one another” [22]. The language of individualism is the most prominent expression of moral life in such a way that the articulation of the second language, the common good of the body politic, becomes quite hard [17]. The sociopolitical underpinnings of public health emphasize community values. This language is obviously not the dominant one in contemporary USA where the emphasis placed on the individual makes it very difficult to appeal to the common good. However, in the USA, one can find active tradition that emphasizes interdependence and communality which contradict individualism as a moral ideal [22].

The economic expression of this rejection of the common good is market individualism which is defined as confidence in the free market as a regulatory mechanism and as a belief in the importance of allowing individuals to make choices within the market and to accept responsibility for consequences that arise from them [25]. Market individualism has been so dominant that the call to individual responsibility has overshadowed the power of collective agency. The market imposes limits on the state’s intervention in individual pathology and welfare, and thus favors the growth of unjustifiable inequalities that weaken the cohesiveness of society and validate, implicitly or not, racism and socioeconomic exclusion. It is, thus, regrettable that an important public health issue such as the access to healthy food would be discussed among some policymakers and intellectuals “exclusively within the dominant discourse of political individualism, relying either on the harm principle of Mill or a narrow paternalism justified on grounds of self-protection alone [22]”. The communitarian language of constitutional republicanism, which reflects the language of public health, is important for communal life because health and safety are a signal commitment of the common life—a central practice by which the body-politic defines itself and affirms its values [17,22].

We justify our recourse to the common good on the basis that market individualism has failed as a model for food security and health policy [25]. The notion that consumers make free and informed choices is not always true because factors beyond persons’ control shape the food environment and determine consumers’ choice [25]. Unlike market individualists, government can intervene to reshape the food environment and to reduce the economic and the human cost of food insecurity. Intervention in the market is justified by the fact that the lack of access to quality food creates social and health costs that other citizens must bear. Intervention is warranted when the common good is at stake. Instead of individualism, which often sustains the logic of the market, solidarity is the way forward. Far from being a mere expression or attitude of condescending charity, solidarity refers to the institutional solutions and interventions designed and implemented by the state or federal authorities to attend to the needs of individuals or population groups who are facing quality food challenges. As a social virtue, solidarity opposes socioeconomic exclusion through which individuals or population groups are often left by their community to bear terrible burdens alone [17]. Solidarity requires state intervention to prevent suffering wherever it is possible and to monitor the activities of greedy market strategists.

#### Market individualism and food system

The promotion of equity in access to quality food demands a shift from a food system which is exclusively market-based to one which is justice-based. Such an approach to food access requires a population-based understanding of justice that requires justice not only in the food domain but also in other domains of social life. As a country that has publicly pledged to promote the human right to food, the federal government needs to carry this notion into action by creating national governing bodies that will actively address the setbacks in each sector of production, consumption, distribution and transportation to ensure food security. Surprisingly, the US government tends to favor the market at the expense of people [25]. Even though there is a growing opposition to the power of food corporations; the food system has not changed. This inertia reflects the liberal understanding of the common good that shapes economic and political decision-making in the country. The US policy environment reflects a preference for markets over government intervention to prevent and to reduce controllable risks [25]. A market-driven mentality has excluded other conceptions of political life, with access to funds widely defining political influence [25]. A market model of competition, bargaining, and profit winning defines the new norms of political conduct. Developing policies that support the market may compromise initiatives in favor of health promotion. Social justice is advanced through activities intended to alter dynamics that affect the common good by modifying social structures and institutions to achieve more democratic and equitable opportunities and outcomes in the distribution of dimensions of well-being.

Accurate food labeling ensures consumers’ right to know about issues that may affect them and promotes informed decision-making in food purchasing. The food industry and corporations should not only keep people nutritionally informed but should produce healthy food in order to promote consumers’ health. Producing, promoting or selling food that is known to be destructive to human health is ethically problematic. To name a social or market practice as unethical or vicious is in a way to assert the need for change due to potential harms that such a practice may cause to individuals or society. Unfortunately, primacy seems to be given to profit generated by the capital and not to human beings. Targeting poor or vulnerable population groups as potential consumers of health-damaging food is ethically vicious. Children, a vulnerable population group, are an important target of the food industry. Regulations that aim at limiting the advertising of unhealthy foods and beverages to children in a variety of media should be developed by the national government. Other measures to limit the promotion of unhealthy foods, such as refusing offers from food and beverage companies to sponsor equipment and programs in schools, are also important [54]. As an institution functioning within society, the food industry has the moral obligation to promote public health. Food corporations should refrain from advertising and selling products that affect the human health.

Although “the food industry has already begun taking many of these actions and should continue to do so in the future” [23], government officials need to monitor the achieved gains and to ask for more. The call for more responsibility is evidenced by the 2007 American Public Health Association policy statement on food system sustainability in which it is stated that “larger agricultural, processing, and retailing companies dictate food prices; influence public policy; control information; and determine the choices and risks available to consumers, food producers, and other workers” [55]. American public health experts furthermore note that “food system impacts are unequally distributed, with the greatest costs (health effects and health-related costs, low wages, stressful conditions) borne by food producers and other workers, rural communities, and low-income consumers. Taxpayers also support this system through health care, social services, infrastructure, and subsidies and other benefits that accrue disproportionately to the largest agri/food businesses” [55].

As a prime promoter of public health, the government should continuously monitor food corporations to ensure that they properly inform the public about food quality [23]. Adequate monitoring will ensure that access to healthy nutritional food will help to facilitate healthy food choices. Federal and state officials should monitor the marketplace so as to eliminate deceptive and greedy practices. It is worth mentioning that “Various government agencies, such as the Food and Drug Administration and the US Internal Review Service, have already taken action” [23]. The European Union (EU) has developed a health policy framework that focuses on the determinants of health and places governmental responsibility at the center of health promotion. The EU health policy focuses on the social determinants of health, in particular as they affect the health divide in Europe, as well as the social gradient in individual societies and in vulnerable population groups. This policy addresses other determinants of health, such as lifestyles, the environment and climate change, and food safety [56]. The EU health policy understands health promotion as a governmental responsibility and emphasizes the need for inter-sector collaboration, while maintaining the important and critical role played by the health minister, who should take the lead for all health-related matters within and beyond the health care system [56]. A close analysis of the EU health policy shows that governmental intervention in the health and health-related spheres, such as access to nutritious food, should not be considered as a dictatorial intrusion in the marketplace because the market does not seem to promote public health and to ensure a successful access to quality food [56]. The analysis of the EU policy shows that the values and beliefs of the public health community often stand in contradiction to the libertarian thinking that shapes the dominant values of the market in the USA. Advertisement to children of foods high in sugar and fat represents an example of this discord. Faced with such an assault on public health, the government cannot help but strengthen its regulatory role in order to protect vulnerable individuals.

#### Policy and advocacy initiatives for healthier eating

Since cost and availability are cited as two important barriers to healthy eating [54], lifestyle change initiatives and health education may not be completely effective in increasing healthy food consumption if it does not take into consideration neighborhood segregation, market strategies, retailer competition, poverty, and major forms of social discrimination as important modifiers of accessibility. A recent USA survey has demonstrated that there has not been any increase in fruit and vegetable consumption from 1988 to 2002 despite a fruit and vegetable campaign initiated in 1991 [32]. Some studies have also demonstrated that these nutritional interventions are less effective among those of lower socioeconomic status [57,58], likely due to a combination of socioeconomic barriers and neighborhood segregation. Thus, health education intervention needs to be combined with a macro-level policy that will address the impact of advertising on consumer choice and will change the economic environment that destabilizes food supply and influences food accessibility in low-income neighborhoods.

Healthy food access is often influenced by several factors, including affordability, availability, food environment, media/advertising, in addition to health education. Affordability refers not only to income, but also the price of healthy foods in relation to unhealthy foods, the price of services that influence the price of healthy foods (such as transportation), and external factors (taxes, subsidies). Several studies have demonstrated that manipulation of cost can lead to increased healthy food consumption [59-61]. For example, experiments that directly reduced the prices of lower-fat snacks by 10%, 25%, and 50% in vending machines resulted in an increase in the sales of these snacks by 9%, 39%, and 93%, respectively [60].

The federal and local governments should address all forms of socioeconomic discriminations and social inequities that prevent individuals and families from living a decent life. Possible policy options would consist of raising minimum wage, providing greater tax benefits or income supplements, and increasing welfare assistance. These policy proposals require further research, since their effectiveness to increase healthy food consumption remains unknown. There are also concerns that raising minimum wage may increase payroll costs and thereby increase the price of goods such as healthy food, as well as increase income taxes paid by low-income workers, perhaps precluding welfare or other benefits and thus, ultimately, decreasing disposable income for food purchases.

More recently, policy proposals have focused on “fat” taxes and “thin” subsidies as a means to promote healthy food consumption, whereby fat taxes refer to sales taxes imposed on unhealthy foods while thin subsidies refer to subsidies for healthy food consumption. Although small to moderate taxes on “snack” food may negatively affect consumption, the resulting health gains may be moderate, estimated at almost 1500 deaths from coronary heart disease (CHD) prevented [62]. The primary objection to this policy is grounded in equity, since fat taxes may disproportionately affect low-income individuals who often spend a greater proportion of their income on food [63-65]. The definition of snack foods that qualify for this type of taxation is also blurred – is a granola bar a unhealthy snack that requires taxation? Currently, a number of countries impose taxes on unhealthy foods such as soft drinks, candy bars, and chips. However, revenue from these taxes is not used solely to subsidize healthy foods. The impact of this policy intervention depends on the size of the subsidy and the consumer response to price changes, which, in turn, depends on the price elasticity of demand. Thus, to be effective, the size of a subsidy must take into account the price elasticity of demand [66]. Simulation analysis of thin subsidies has revealed that a 1% price decrease in all fruits and vegetables can prevent a total of 9680 cases of CHD and ischemic stroke, but the benefits are lowest among low-income compared to high-income individuals (2668 vs. 4834 cases prevented) [67]. Although subsidies do not demonstrate the “regressiveness” of fat taxes, subsidies alone will not suffice to improve healthy food consumption, particularly in low-income households.

Beyond affordability and retail competition, healthy food consumption also depends on consumer choice and availability. Availability is influenced by cultural preferences, health education, retail competition, geographic consideration and advertising/media. Policy proposals to increase advertising of healthy foods and to limit advertising of unhealthy foods during peak television viewing hours may be beneficial to reducing unhealthy food consumption. Within the food environment, promotion of local market efforts such as farmers’ markets through governmental incentives and increased law enforcement where crime precludes the establishment of grocery stores may ameliorate accessibility. Several provincial governments already regulate the price of alcohol/tobacco but do not do the same for healthy foods. While many would suggest caution with respect to increasing the presence of local government intervention in food access and marketing, the US government already collaborates with marketing boards such as dairy and egg farmers to help ensure quality, affordability, and availability of these products. The current discrepancies in food accessibility may encourage local governments to expand their market regulatory role in partnership with local communities, food manufacturers, retailers and marketing boards.

In order to promote healthy eating, the US Department of Agriculture (USDA) changed the food pyramid to the plate method. The Plate Method was originally developed as a way of teaching people with diabetes about portion control and what to eat at each meal [68]. The Plate Method of eating has gone on to become an easy way to teach adults and children about how to eat the right amount of foods from different food groups. The Plate Method is a way to visually control one’s diet [68]. According to the American Diabetes Association the breakdown of food on your plate should include: “50% of vegetables, 25% of carbohydrates, 25% lean protein; one serving of milk, yoghurt or calcium; and one serving of fruit” [69]. This shows that significant efforts have been deployed by the US government and other social constituencies to promote healthy eating. This change addresses the issue of quality but does not address the issue of accessibility to the type of food required for good health. Access strategies aiming at improving food quality and consumption have been developed and implemented in Pennsylvania, New York and Los Angeles [69]. These strategies include increasing the availability of supermarkets and corner stores selling healthier foods; promoting institutional procurement policies to increase healthier foods at work sites; supporting farm-to-institution programs, and limiting the availability of high-energy dense foods and sugar-sweetened beverages [69]. Poor and low-income individuals are the prime targets of these interventions. In Pennsylvania, the Fresh Food Financing Initiative, for example, targets underserved areas for which funds are provided to supermarkets in order to improve access to health foods [23]. In the same vein, “the Healthy Bodegas Initiative of New York City has funded local bodegas to expand the availability of healthier food choices throughout the metropolitan area” [23]. In the city of Los Angeles, “a one year moratorium on the development of new fast-food establishments within a neighborhood of thirty-two square miles of poor and low-income residents restricts access to less healthful products by using zoning regulations” [23].

To increase healthy food consumption, there is a need for partnership and advocacy on several fronts – government, food manufacturers, retailers, marketing boards, media, health professionals, and local communities. One of the major functions of any government is to ensure public safety and protect public health. To address disparities in healthy food consumption, both federal and state officials can play an important role. They have to develop and implement policies focused on favoring the social inclusion of low-income population groups, challenging neighborhood segregation and addressing other forms of social inequities. To achieve these goals, both federal and state government would expand their partnerships with marketing boards, transportation providers, and food manufacturers to increase availability while limiting increase in cost of healthy foods. At the state level, governments can work with stakeholders and local communities to improve neighborhood safety, and to promote additional sources of healthy foods. Working in partnership with local communities, health professionals, and media to promote culture-specific and multilingual health education efforts can improve accessibility. Community agency plays an important role in mobilizing residents to join coalitions with health professionals and other constituencies to collect evidence and lobby their elected officials and legislators to improve healthy food consumption.

Journalists can play a key role in increasing awareness of the disparities in healthy food consumption, stirring up local communities and forging partnerships among the various groups, including groups of health professionals. Individually, health professionals can act as resources for educating and increasing awareness in patients and communities, monitoring food insecurity, and providing information to policy-makers. Collectively, medical associations can lobby policy-makers and national stakeholders for increased awareness and monitoring of disparities in healthy food consumption on a population level, advocating for health insurance coverage of nutrition counseling as part of “preventive” health efforts, working with urban planners to ensure favorable neighborhood characteristics for food retail establishment, urging government initiatives to provide incentives to food manufacturers for the development of healthy food alternatives, and increasing research with policy-makers and economic analysts regarding the potential benefits of policy changes on disease outcomes. As a critical mass, health professionals can act as attorneys of the marginalized, transcending mere medical solutions to argue for structural change [70].

#### Empowerment, advocacy and community agency

Public health emphasizes the interconnection among human beings in a democratic society as well as the importance of individual and community participation in health promotion initiatives. The improvement of people’s participation is a basic requirement of democratic governance. Participation is a basic dimension of well-being that every citizen should, in one way or another, enjoy. All in society must have both the right and the capacity to participate effectively in defining what is common to members of the community—and what is not—through discourse, and action. All citizens should have full and equal possibility to participate directly or indirectly in political decision-making. Political participation is important for effective advocacy initiatives. Advocacy initiatives will not work if, at the grassroots, affected people are not empowered to change their situation. Empowerment presupposes effective participation of people affected by the situation that needs to be changed. It also demands the democratic ruling and policing of the food environment. Empowerment aims at facilitating people in taking direct responsibility for themselves so that they resort to state assistance only where necessary. Genuine activism on behalf of affected individuals or population groups enables them to become part of the solution, as has happened with anti-retroviral treatment campaigns.

To improve access significantly, there is a need to address issues of individual and community agency, because initiatives aiming at distributing food to hard-to-reach or low-income population groups do not suffice. Very often, approaches to distributive justice focus on commodity distribution based on the concept of basic needs. Addressing the social and political environment of food accessibility requires that one goes beyond the immediate need for healthy food to identify lasting strategies for change. The point is to look for ways of changing power configuration so that victims of food discrimination can actually act, at least at the local level, upon the causes that prevent them from having access to healthy food. Our approach to empowerment does not focus on actual functionings, such as healthy consumption or health care delivery, but on capabilities [71,72] or dimensions of well-being [37]. Whether we focus on capabilities or dimensions of well-being in relation to healthy food accessibility, we argue that it is important to refer to conditions that make it possible for poorly malnourished individuals or population groups to have adequate access to food.

Interventions that have been most integrated with economic, education, and/or political sectors have resulted in greater psychological empowerment, autonomy and authority, and have substantially affected a range of health outcomes [73]. For example, multi-level empowerment strategies for HIV/AIDS prevention which address gender inequalities have improved health status and reduced HIV infection rates [73]. Furthermore, women’s collaborative empowering interventions in the economic, educational, and political sectors have shown the greatest impact on women’s quality of life, autonomy and authority and on policy changes, as well as on improved child and family health. Similarly, empowering affected people will increase their abilities to manage their environment, to adopt healthier behaviors, to use health services more effectively, and to advocate for their needs [73]. Coalitions and inter-organizational partnerships in affected neighborhoods or communities can promote empowerment through enhanced participation and environmental and policy changes. To increase chances for locally-grounded actions, federal governments need to support and encourage the work of subsidiary bodies.

## Conclusion

When we use the common good to address the accessibility to healthy food in the USA, we move away from focusing on the individual access to food and on individual vulnerability to poor diet to a focus on the social determinants of access. The common good brings about connections between single entities and social networks on the one hand, and individuals and social institutions on the other. We then reject moral and social individualism as potential analytical framework. As an analytical tool, individualism cannot account for the social roots of poor diet and food insecurity. Instead, the common good leads us into the search for both the immediate and the distal causal factors of healthy food inaccessibility at the population level. In contrast to the reliance on moral and social individualism, the common good broadens our moral compass and expands our moral imagination by challenging us to focus on social relationships and policies that determine accessibility to healthy food. Thus, we focus on social practices and appeal to forms of partnerships that can alter the dominant mindsets and practices that create inequity in access.

The more we use the language of the common good to understand the unequal access to healthy food and the related public health consequences, the more we can expand our agenda to other social issues, our circle of interlocutors and our moral discourse. The common good has a community emphasis that connects well to the public health emphasis on population. Public health identifies and measures threats related to the health of communities rather than individuals, develops policies in response to those concerns and is more focused on preventive interventions. Public health seeks to minimize threats to population health that can be lessened only through collective actions. Both the public health perspective and the common good ethics give pre-eminence to communal goals over individual goals without neglecting the latter. As a public health threat, healthy food inaccessibility cannot be solved by individuals’ efforts or by implementing libertarian policies. Effective changes incorporating this common good approach entails more than exhortations to care for each other. Fundamentally, it entails the radical modification of the socioeconomic and political environment that shapes both food distribution and access to food across the USA population. We need to involve many constituencies and to uphold a population-based approach to justice because the common good is a bridge-building device.

Although the importance of diet to reduce the chronic disease burden is well-established, social and economic barriers often preclude healthy food consumption, particularly in socially or geographically marginalized populations. The common good argument requires that policy decision, including food policy, be made on the basis of people’s well-being. The lack of quality food seems to be primarily a matter of social justice and human rights because this phenomenon is essentially rooted in social structures. Efforts to increase healthy food consumption solely through health education efforts and food distribution may have been shown not to suffice. Research into the development and implementation of policies that focus on ameliorating societal inequities, food environment, consumer choice, and retail competition is warranted. Importantly, advocacy and partnership among a variety of agents including health professionals is required to address this important public health issue.

## Competing interests

The authors declare that they have no competing interests.

## Authors’ contributions

JA and TJ originated the article, did the research and wrote a first rough draft of the manuscript together. TJ did the research on the health consequences of poor eating and JA carried out the research on ethical and policy aspects. JA and TJ all read the first version and contributed editorial and critical suggestions. After the first peer-review, JA made revisions to the earlier draft and worked toward the final draft. They have both read and approved the final version of the manuscript.

## Authors’ information

Jacquineau Azétsop lectures health policy, medical deontology and bioethics at Faculté des Sciences Médicales de l’Université de N’djamena in Chad. He also teaches public health and population-level bioethics in the Master of Public Health and HIV/AIDS management at the Catholic University of Mozambique in Beira. Jacquineau has published several articles in the fields of public health ethics, bioethics and public health.

Dr. Tisha Joy is an assistant professor at the Division of Endocrinology and Metabolism, Department of Medicine of Western Ontario University. Her clinical and research interests are in cholesterol disorders, obesity, and type 2 diabetes. She has published several articles in the fields of metabolic syndrome, body fat distribution, and cholesterol disorders.
